# PI3Kp110-, Src-, FAK-dependent and DOCK2-independent migration and invasion of CXCL13-stimulated prostate cancer cells

**DOI:** 10.1186/1476-4598-9-85

**Published:** 2010-04-22

**Authors:** Christelle P El Haibi, Praveen K Sharma, Rajesh Singh, Paul R Johnson, Jill Suttles, Shailesh Singh, James W Lillard

**Affiliations:** 1 Department of of Pathology, Beth Israel Deaconess Medical Center, Harvard Medical School, Boston, MA, USA; 2 Department of Microbiology & Immunology, University of Louisville School of Medicine; Louisville, KY, USA; 3 Department of Microbiology, Biochemistry & Immunology, Morehouse School of Medicine, Atlanta, GA, USA; 4 Department of Biochemistry & Molecular Biology, University of Louisville School of Medicine; Louisville, KY, USA

## Abstract

**Background:**

Most prostate cancer (PCa)-related deaths are due to metastasis, which is mediated in part by chemokine receptor and corresponding ligand interaction. We have previously shown that PCa tissue and cell lines express high levels of the chemokine receptor CXCR5, than compared to their normal counterparts, and interaction of CXCR5 with its specific ligand (CXCL13) promoted PCa cell invasion, migration, and differential matrix metalloproteinase (MMP) expression. This study dissects some of the molecular mechanisms following CXCL13-CXCR5 interaction that mediate PCa cell migration and invasion.

**Results:**

Using Western blot analysis, kinase-specific cell-based ELISAs, and migration and invasion assays, we show that PCa cell lines differentially express phosphoinositide-3 kinase (PI3K) catalytic subunit isoforms and dedicator of cytokinesis 2 (DOCK2). Specifically, we show that PC3 and normal prostatic epithelial (RWPE-1), but not LNCaP cell lines expressed DOCK2, while RWPE, PC3, and LNCaP cell lines expressed PI3K-p110α and -p110β. Moreover, PC3 selectively expressed PI3K-p110γ, but LNCaP and RWPE cell lines expressed PI3Kp110δ. CXCL13 caused CXCR5-dependent activation of the PI3Kp85α in LNCaP cells, and p85α as well as -p101 in PC3 cells. CXCL13-CXCR5 interaction regulated LNCaP and PC3 cell migration and invasion through extracellular signal-regulated kinase 1/2 (ERK1/2) activation that was primarily dependent on the PI3Kp110 isoform(s), Src, and focal adhesion kinase (FAK), but not DOCK2.

**Conclusions:**

While additional studies will be needed to determine the PI3K-independent (i.e., DOCK2-mediated) and -dependent events that dictate PCa cell responsiveness to CXCL13, these data provide evidence of the existence of cell type- and stimulus-specific signaling events that support migration and invasion of PCa cells.

## Background

PCa is the second most commonly diagnosed cancer in men after skin cancer [1,2]. Increased public awareness and advances in diagnostic tools have helped detect this disease at an early stage, i.e., when the tumor is localized to the prostate gland. Unfortunately, 2.5% of patients will suffer from metastasis and eventually die from associated complications [3]. Patients with advanced PCa initially respond to hormone therapy to decrease testosterone levels, but often develop refractive tumors. In addition, and for yet not fully defined reasons, this advanced stage (hormone refractory) is associated with high incidences of PCa spread to bones [4-6]. It is thought that the bone microenvironment composition (e.g., mineralized bone matrix, growth factors, etc.) and its physical properties (e.g., hypoxia, acidic pH, extracellular calcium, etc.) provide a favorable milieu for tumor invasion and growth [7-9].

Malignant cells exhibit aberrant expression of particular chemokine receptors relative to their normal counterparts [10-15]. We have recently shown that prostate carcinomas differentially express CXCR5 and its expression positively correlates with stage and grade [16]. CXCR5 is a seven transmembrane G-protein coupled receptor for the chemokine CXCL13. The CXCR5 gene is specifically expressed in Burkitt's lymphoma and lymphatic tissue and plays an essential role in B cell migration. We demonstrated that CXCR5-bearing PCa cell lines selectively express certain MMP in response to CXCL13 [16-18]. One means by which the bone microenvironment is thought to recruit PCa cells is through bone expression of CXCL13 [19]. Thus, by virtue of its presence in the bone microenvironment, we hypothesized that CXCL13-CXCR5 interactions help to regulate PCa cell migration and invasion.

LNCaP and PC3 cell lines are extensively used models to study cell signaling that may occur during PCa progression [20,21]. LNCaP cells are androgen-dependent and express prostate specific antigen (PSA), whereas PC3 cells are androgen-independent and are unable to secrete PSA. The acquired hormone-refractory properties have been linked to the high skeletal metastatic potential of PC3 cells compared to a lower potential of the hormone-responsive LNCaP cells. These and other differences allow LNCaP and PC3 cell systems to provide meaningful insights into specific cellular events involved in PCa spread to bones. In this study, we use LNCaP and PC3 cell lines to elucidate the differences in CXCR5-mediated signaling related to cell migration and invasion, compared to a normal prostatic epithelial cell line (RWPE-1).

PI3K(s) are central signaling molecules activated through chemokine receptor-mediated signaling [22]. Chemokine receptors are coupled to heterotrimeric G proteins α, β, and γ, which subsequently activate Class IA and IB PI3Ks, respectively. Class IA PI3Ks consist of three catalytic isoforms - p110α, p110β, and p110δ, which associate with a p85α regulatory subunit, whereas Class IB PI3Ks are comprised of p101 regulatory and p110γ catalytic subunits. Following activation, PI3K catalyzes the conversion of phosphoinositide 4,5-biphosphate (PIP2) to generate phosphoinositide 3,4,5-triphosphate (PIP3) [23-27]. This reaction can be counterbalanced by the action of the lipid phosphatase and tensin homolog deleted on chromosome ten (PTEN). However, PTEN is frequently lost in PCa leading to accumulation of PIP3, which activates Akt- and ERK-dependent signaling leading to enhanced cell migration and invasion [28-30]. On the other hand, DOCK2, a novel member of the Caenorhabditis elegans Ced-5, mammalian DOCK180, and Drosophila melanogaster MyoblastCity (CDM) family of scaffold proteins, has been shown to regulate cytoskeletal dynamics by activating Rac isoforms [31,32] and directing lymphocyte and neutrophil chemotaxis [33-35]. To our knowledge, the role of DOCK2 in PCa progression, and specifically in tumor cell migration and invasion, has not been studied. In addition, PCa cell invasion is a complex process, which also includes changes in cell adhesion mediated in part through the FAK and Src [36-39]. Indeed, aberrant FAK and Src activation has been correlated with increased tumor growth and metastasis following chemokine-mediated signaling. Here we report on the role of PI3K, DOCK2, Src, and FAK in PCa cells and reveal some of the molecular mechanisms of CXCL13-CXCR5 interaction that mediate PCa cell invasion and migration.

## Results

### Differential expression of PI3K isoforms and DOCK2 by RWPE-1, LNCaP, and PC3 cell lines

**Figure 1:**
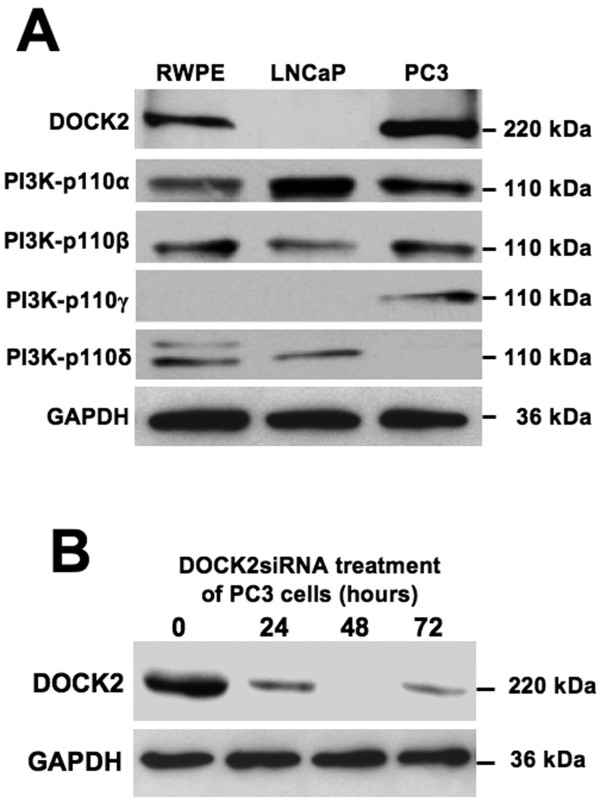
**Differential expression of PI3K catalytic isoforms and DOCK2 by PCa cell lines**. **(A) **Total cell lysates (60 μg) from RWPE-1, LNCaP, and PC3 cells were resolved by SDS-PAGE and subjected to immunoblotting using antibodies against PI3Kp110α, p110β, p110γ, p110δ, and DOCK2 (Santa Cruz). GAPDH detection served as a loading control. **(B) **DOCK2 silencing conditions were optimized by transfecting PC3 cells with 1 μg of DOCK2 siRNA duplex incubated cells for 0, 24, 48, and 72 hours. The efficacy of DOCK2 silencing was determined by Western blot analysis.

**Figure 2:**
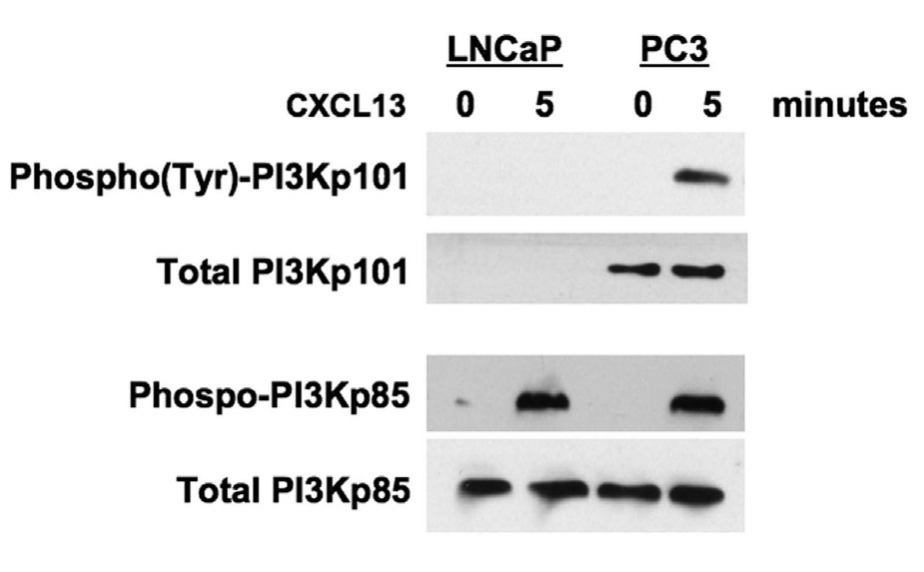
**Differential expression and activation of PI3K regulatory isoforms by LNCaP and PC3 cell lines**. LNCaP and PC3 cells were treated with CXCL13 for 0 or 5 minutes. Cell lysates were subjected to immunoblotting with antibodies against phospho-PI3Kp85α or phospho-tyrosine following immunoprecipitation with anti-PI3Kp101 antibody.

RWPE-1 and LNCaP cell lines expressed PI3Kp110α, p110β, and p110δ, while PC3 cells expressed PI3Kp110α, p110β, and p110γ isoforms (Figure 1A). DOCK2 was present in RWPE-1 and PC3, but not LNCaP cell lines. To verify the specificity of the DOCK2 siRNA, we treated PC3 cells with 1 μg siRNA and immunoblotted for DOCK2. Optimal silencing of DOCK2 occurred after 48 hours (Figure 1B). To demonstrate CXCL13-mediated regulation of PI3K, we used phosphorylated PI3K regulatory subunit as an indicator of PI3K activation, as previously described by Cuevas *et al. *[40]. LNCaP and PC3 cell lines showed differential CXCL13-mediated activation of PI3K regulatory subunits. CXCL13 activated only PI3Kp85α in LNCaP cells, but phosphorylated both PI3Kp85α and p101 subunits in PC3 cells (Figure 2). Together, our data show differential expression and phosphorylation of PI3K isoforms and DOCK2 by RWPE-1, LNCaP, and PC3 cell lines following CXCL13 stimulation.

### PI3K-, Src-, FAK-dependent, and DOCK2-independent PCa cell migration and invasion

**Figure 3:**
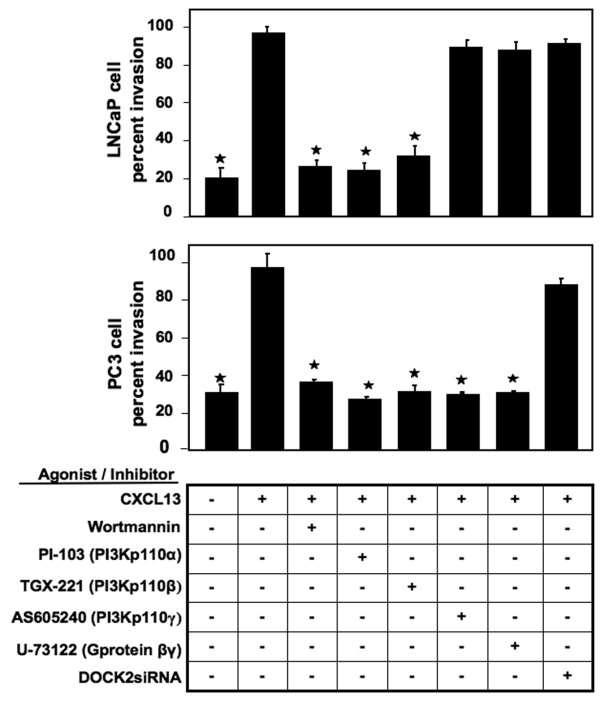
**PI3K regulation of PCa cell migration and invasion**. LNCaP and PC3 cell lines were tested for their ability to invade Matrigel™ Matrix and migrate through an 8.0 μm porous membrane in the presence of CXCL13 and/or wortmannin, PI-103, TGX-221 and AS605240, U73122, or DOCK2 siRNA. Cells that invaded or migrated to the lower surface of the membrane were stained with crystal violet and counted by microscopy at 40× magnification. Percent cell invasion was calculated following manufacturer's instructions (BD Biosciences). Asterisks indicate significant differences (p < 0.05) with CXCL13-treated cells.

**Figure 4:**
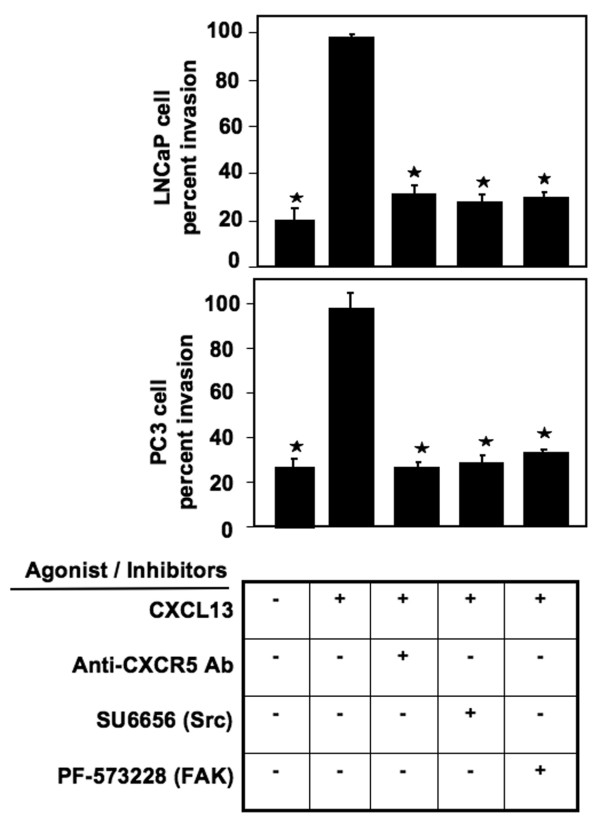
**Role of Src and FAK in PCa cell migration and invasion**. LNCaP and PC3 cell lines were tested for their ability to invade Matrigel™ Matrix and migrate through an 8.0 μm porous membrane in the presence or absence of CXCL13, anti-human CXCR5 antibody, SU6656, and PF-573228. Cells that invaded or migrated to the lower surface of the membrane were stained with crystal violet and counted by microscopy at 40× magnification. Percent cell invasion was calculated following manufacturer's instructions (BD Biosciences). Asterisks indicate significant differences (p < 0.05) between CXCL13-treated and untreated cells.

To determine whether activated PI3Ks, Src, and FAK promoted invasiveness of PCa cells, we used corresponding pharmacological inhibitors and assessed their effect on cell invasion. As expected, RWPE-1 cells were completely noninvasive in response to CXCL13 (data not shown), while LNCaP and PC3 cells were invasive (Figure 3). Wortmannin, PI-103 (PI3Kp110α inhibitor), and TGX221 (PI3Kp110β inhibitor) significantly reduced CXCL13-mediated LNCaP and PC3 cell migration and invasion. While PI3Kp110γ inhibition abrogated the ability of PC3 cells to migrate and invade, it did not affect the motility and invasiveness of LNCaP cells. Similarly, U-73122 (G protein β and γ inhibitor) impaired the ability of PC3, but not LNCaP, cells to migrate and invade. Treatment of LNCaP and PC3 cells with DOCK2 siRNA had no effect on cell invasion. These findings show that CXCL13-mediated LNCaP cell migration and invasion is PI3Kp110α- and p110β-dependent, whereas PC3 cell migration and invasion is PI3Kp110α-, p110β-, and p110γ-, and G protein β- and γ-dependent. Src and FAK are also key molecules involved in chemokine-mediated signaling and promote tumor growth and metastasis [36,39]. Src, FAK, and CXCR5 inhibition significantly impaired PCa cell migration and invasion in response to CXCL13 (Figure 4). This suggests that the Src-FAK axis also plays a role in CXCR5-mediated PCa cell migration and invasion.

### ERK1/2 activation by CXCL13-treated PCa cells

**Figure 5:**
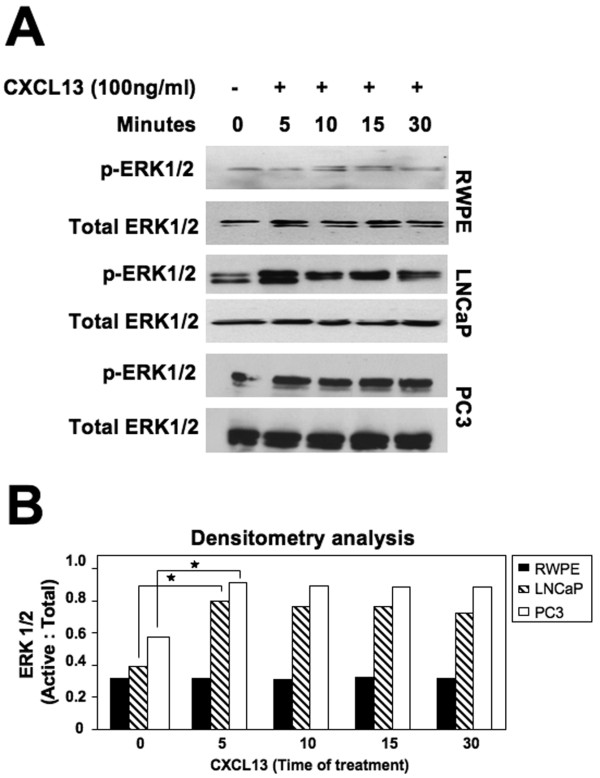
**ERK1/2 Activation by CXCL13-treated PCa cells**. **(A) **RWPE-1, LNCaP, and PC3 cell lines were serum starved, treated with 100 ng/ml CXCL13 for 0, 5, 10, 15 and 30 minutes, and lysed. Cell lysates were subjected to immunoblotting with antibodies against p-ERK1/2. Membranes were stripped and reprobed with antibodies to total ERK1/2. **(B) **Densitometry analysis was performed using VersaDoc Imaging System (Biorad). Histograms show the differences in ERK1/2 levels between LNCaP and PC3 cells at basal activity and after CXCL13 stimulation. Asterisks indicate significant differences (p < 0.05).

G-protein coupled receptors can lead to ERK1/2 signaling cascades [41]. Active (phosphorylated) levels of ERK1/2 remained relatively low in RWPE-1 cells treated with or without CXCL13. LNCaP cells showed reduced basal (i.e., time 0) levels of p-ERK1/2, but significant increases in phosphorylated (p-) ERK1/2 levels > 5 minutes after CXCL13 stimulation. PC3 cells, on the other hand, had elevated basal levels of p-ERK1/2, which were significantly elevated after CXCL13 addition (Figure 5). These findings in part support the greater ability of PC3 cells to invade ECM than compared to LNCaP cells. Since CXCR5 is expressed by PCa cells but not by normal prostate cells, our findings also suggest that CXCL13-CXCR5 interaction regulate ERK1/2 phosphorylation in PCa (LNCaP and PC3) cells, but not in normal prostatic epithelial (RWPE) cells.

### PI3K-, Src-, FAK-dependent, and DOCK2-independent ERK1/2 regulation by PCa cells

**Figure 6:**
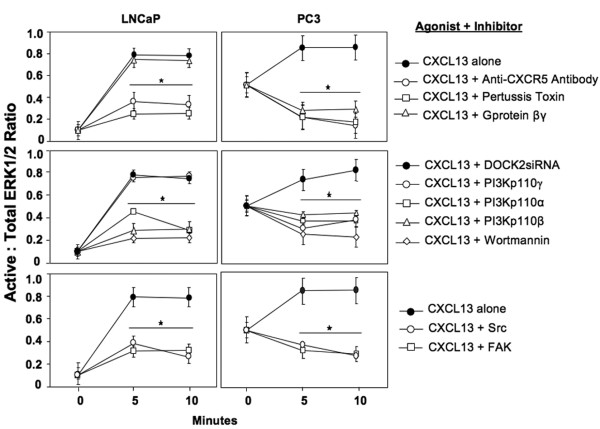
**CXCL13-CXCR5 signaling events required for ERK1/2 activation**. FACE assays were performed to measure active and total ERK1/2 levels in LNCaP and PC3 cell lines. Cells were treated with (or without) CXCL13 for 5 or 10 minutes, along with or without CXCR5 blockade, pertussis toxin, U-73122, wortmannin, PI-103, TGX221, and AS605240, DOCK2 siRNA, SU6656, and PF-573228. Experiments were performed in triplicate and results show the ratio of p-ERK1/2 to total ERK1/2.

To delineate CXCL13-CXCR5 signaling events required for ERK1/2 activation in LNCaP and PC3 cells, we performed a ERK1/2-specific fast activated cell-based ELISA (FACE) assay in the presence of various PI3K isoform inhibitors, DOCK2 siRNA, pertussis toxin, G protein β and γ inhibitor, PF-573228 (FAK inhibitor), and SU6656 (Src inhibitor). CXCL13-treated LNCaP cells exhibited an eight-fold increase in p-ERK1/2 to total ERK1/2 ratio, compared to untreated cells (Figure 6). Treatment with CXCR5 blockade or pertussis toxin significantly abrogated CXCL13-mediated ERK1/2 activation. However, G protein β and γ inhibition did not have an effect on ERK1/2 activation following CXCL13 stimulation of LNCaP cells. Treatment of LNCaP cells with wortmannin, PI3Kp110α, PI3Kp110β, FAK, or Src inhibitors lead to a significant reduction in ERK1/2 activation indicating that PI3Kp110α, PI3Kp110β, FAK, and Src play a role in LNCaP CXCL13-mediated ERK1/2 signaling. On the other hand, PI3Kp110γ inhibition did not influence ERK1/2 activation, suggesting CXCL13-mediated ERK1/2 activation is PI3Kp110γ-independent in LNCaP cells. As expected, DOCK2 siRNA had no effect on ERK1/2 activation in LNCaP cells, as these cells lack DOCK2.

CXCL13 increased the ratio of p-ERK1/2 to total ERK1/2 in PC3 cells. These ratios were significantly reduced in PC3 cells following CXCR5 blockade or treatment with pertussis toxin or G protein β/γ inhibitor, suggesting CXCR5-mediated ERK1/2 activation can be regulated through G protein β and/or γ subunits in response to CXCL13 stimulation. Treatment of PC3 cells with wortmannin, PI3Kp110α, p110β, p110γ, FAK, and Src inhibitors lead to a significant reduction in p-ERK1/2 indicating that PI3Kp110's, FAK, and Src promote CXCL13-mediated ERK1/2 activation. In contrast, DOCK2 siRNA-treated PC3 cells showed comparable levels of p-ERK1/2 to total ERK1/2 as cells treated with CXCL13 alone, suggesting that CXCL13-mediated ERK1/2 activation is DOCK2-independent.

## Discussion

PCa cells aberrantly express CXCR5, which plays a significant role in cell invasion, migration, and differential matrix metalloproteinase (MMP) expression [16,18]. In addition, it is known that metastatic PCa cells favorably migrate to bone [4,5,42,43], which can produce CXCL13. Hence, CXCL13-CXCR5 interaction might enable migration and invasion of PCa cells to bone. LNCaP and PC3 cell lines lack the lipid phosphatase PTEN [30,44]. As a result of PTEN ablation, PIP3 synthesis is deregulated leading to enhanced activation of PI3K signaling, a pathway proposed to play a major role in tumor invasiveness [29,44]. In this study, we show that LNCaP cells express Class IA PI3Kp110α, p110β, and p110δ catalytic isoforms and stimulation of these cells with CXCL13 leads to phosphorylation of the Class IA PI3Kp85α regulatory subunit. In contrast, PC3 cells express the Class IB PI3Kp110γ as well as Class IA PI3Kp110α and PI3Kp110β catalytic subunits and stimulation of these cells with CXCL13 leads to phosphorylation of Class IA PI3Kp85α and Class IB PI3Kp101 regulatory subunits.

Class IA PI3Ks are known to be activated by small G protein α subunit(s), while Class IB PI3Ks are directly regulated by small G protein β and γ subunits [45,46]. To determine the physiological relevance of the different PI3K isoforms expressed by LNCaP and PC3 cells, we performed migration and invasion assays using cell permeable small molecule inhibitors of PI3Kp110α, p110β, and p110γ which function by interacting with the adenosine triphosphate-binding pocket of these enzymes [47-53]. LNCaP cell migration and invasion were regulated by Class IA PI3Kp110α and p110β as well as Src and FAK. Taken together, these results provide one possible explanation for LNCaP cells' reduced invasiveness and inability to metastasize to bone. In contrast, PC3 cell migration and invasion were Class IA (PI3Kp110α, p110β)- and Class IB (PI3Kp110γ)-dependent. PC3 cell motility and invasiveness was also regulated in part by Src and FAK in response to CXCL13. In summary, CXCL13-CXCR5 interaction regulated both Src-, FAK-, and G protein β/γ-dependent signaling cascades, which might contribute to the high metastatic potential of PC3 cells and their ability to spread to bone.

**Figure 7:**
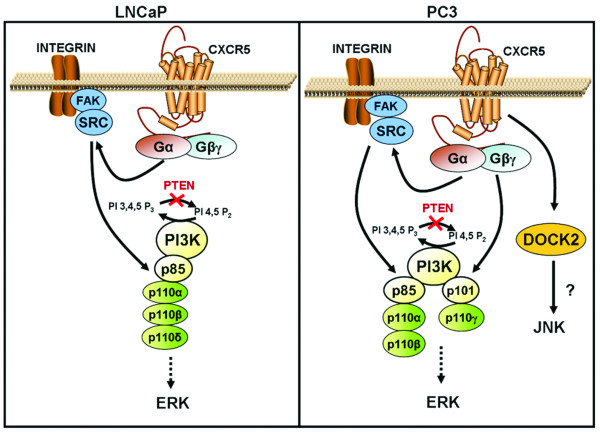
**CXCR5 activate PI3K isoforms through Gα-Src-FAK pathway or via Gβ/γ pathway leading to subsequent activation of ERK1/2**. LNCaP cells express PI3Kp110α, PI3Kp110β and PI3Kp110δ, while PC3 cells express PI3Kp110α, PI3Kp110β and PI3Kp110γ. Perhaps owing to their more aggressive phenotype, PC3 cells are capable of exploiting both Gα-Src-FAK and Gβ/γ-mediated events leading to enhanced migration and invasion, while LNCaP cells are only able to take advantage of the Gα-Src-FAK-mediated pathway following CXCL13-CXCR5 interaction.

Price *et al. *demonstrated that prostate tumors contain elevated levels of p-ERK1/2 in comparison to early-stage or benign specimens [54]. Inhibition of ERK1/2 activation attenuates growth factor-dependent migration and invasion of PCa cells by decreasing MMP expression [55], suggesting a regulatory role for ERK1/2 in PCa metastasis. However, factors responsible for ERK1/2 activation in PCa cells have been incompletely defined. Here, we show a positive role of CXCL13-CXCR5 interaction in eliciting ERK1/2 activation in androgen-sensitive and -independent PCa cell lines. Although basal ERK1/2 activity is more prominent in PC3 cells than in LNCaP cells, the activity of this kinase was significantly higher in both cell lines in response to CXCL13 treatment. ERK1/2 activation in PCa cells was regulated by Class IA PI3K isoforms, Src, and FAK in LNCaP cells treated with CXCL13; however, both Class IA and Class IB PI3K isoforms as well as Src and FAK could lead to ERK1/2 phosphorylation in PC3 cells treated with CXCL13 (Figure 7).

Chemokine-mediated leukocyte migration has also been shown to strongly depend on the Rac activator scaffold protein, DOCK2 [31]. Fukui *et al. *reported that DOCK2-deficient lymphocytes have reduced ability to migrate in response to CXCL12, CXCL13, CCL19, and CCL21 [56]. In this study, we demonstrate that unlike LNCaP cells, PC3 cells express DOCK2. However, DOCK2 inhibition by siRNA did not abolish the ability of PC3 cells to migrate and invade, nor did it modulate ERK1/2 activation.

## Conclusions

These data provide further evidence of the existence of cell type- and stimulus-specific signaling events that support PCa cell migration and invasion. As a downstream target of Rac GTPase, we speculate that Janus kinase - mitogen activated protein kinase (JNK-MAPK), which is known to be induced by CXCL13 [57], could be regulated by DOCK2 to promote PC3 cell proliferation and anti-apoptotic events. Future studies will be necessary to address the precise role of DOCK2 and CXCL13-CXCR5 interaction in PCa progression. While small molecule inhibitors effectively demonstrated the role of PI3Kp110 isoforms in CXCL13-CXCR5 signaling, the use of PI3Kp110 isoform-specific siRNA resulted in lower cell viability in the required time frame (data not shown). Hence, future *in vivo *studies will validate the present findings with inducible PI3Kp110 isoform-specific dominant negative and constitutive active constructs expressed by PCa cell lines as well as expand our investigation of Rac isoform roles in PCa cell proliferation in response to CXCL13. These studies will be essential to completely dissect the PI3K-independent and -dependent (i.e., DOCK2-mediated) events that dictate PCa cell responsiveness to CXCL13.

## Methods

### Cell lines and culture

The RWPE-1 cell line (American Type Culture Collection, ATCC) was established from normal prostate epithelial cells and cultured in keratinocyte serum free media supplemented with bovine pituitary extract (0.05 mg/ml) and epidermal growth factor (5 ng/ml). The LNCaP cell line (ATCC) was derived from the left supraclavicular lymph node of a metastatic prostate adenocarcinoma patient. The PC3 cell line (ATCC) was derived from a bone metastasis from a grade IV prostatic adenocarcinoma patient. PCa cell lines were cultured in complete RPMI 1640 supplemented with 10% fetal bovine serum (FBS) and maintained in a cell culture incubator at 37°C in a humidified atmosphere with 5% CO_2_. All cell lines were serum starved overnight prior to treatment with 0 or 100 ng/ml of CXCL13 (PeproTech) in the presence or absence of: isotype control antibody or anti-human CXCR5 antibody (1 μg/ml, R&D systems), pertussis toxin (100 ng/ml, List Biological Laboratories), G protein β and γ inhibitor (U-73122, 5 μM, Sigma), wortmannin (1 μM, Sigma), small molecule inhibitors of PI3Kp110α (PI-103, 3 μM, Echelon), PI3Kp110β (TGX221, 1 μM, Cayman Chemicals), and PI3Kp110γ (AS605240, 3 μM, Echelon), Src (SU6656, 5 μM, Sigma), FAK (PF-573228, 5 μM, Pfizer), or DOCK2 siRNA (1 μg, Santa Cruz).

### Treatment of cells with siRNA against DOCK2

PCa cell lines were seeded into a 6-well plate at 2 × 10^5 ^cells per well in antibiotic-free normal growth medium supplemented with 10% FBS and incubated until 70% confluency was achieved. Cells were then transfected with 1 μg of DOCK2 siRNA or control siRNA duplex for 6 hours following manufacturer's protocol (Santa Cruz), the growth medium was replaced, and cells were incubated for an additional 24, 48, or 72 hours. The efficacy of DOCK2 silencing was determined by Western blot analysis.

### Immunoblotting and antibodies

Following treatments, RWPE-1, LNCaP, and PC3 cell lines were lysed in buffer containing 50 mM Tris-HCl, pH 7.4, 150 mM NaCl, 1% NP-40, protease and phosphatase inhibitor(s) cocktail (Roche). Protein concentrations of whole cell lysates were determined by bicinchoninic acid (BCA) protein assay (Pierce). To determine PI3Kp101 activation, equal amounts (100 μg) of LNCaP and PC3 cell lysates were incubated at 4°C with 1 μg of anti-PI3Kp101 antibody (Millipore) for 2 hours followed by 20 μl of Agarose A/G PLUS beads (Santa Cruz) overnight. Immune complexes were washed twice with lysis buffer, eluted by boiling in sample buffer for 5 minutes, and subjected to immunoblot analysis.

In general, immunoblot analysis was conducted on immunoprecipitates generated as described above or directly on cell lysates containing 60 μg of protein. Samples were denatured by boiling in Laemmli buffer for 5 minutes, resolved on 4-15% gradient sodium dodecyl sulfate-polyacrylamide gel electrophoresis (SDS-PAGE), and transferred to nitrocellulose membranes using a semi-dry transfer cell system (Bio-Rad). The transfer time varied from 30 minutes to 1 hour depending on the molecular weight of the protein being transferred. Membranes were blocked for 1 hour at room temperature in 5% non-fat milk in 1X Tris-Tween Buffered Saline (TTBS, 30 mM Tris-Base, 150 mM NaCl, and 0.1% Tween 20), followed by washing with 1× TTBS. Primary antibodies against PI3Kp110α, PI3Kp110β, PI3Kp110γ, PI3Kp110δ, DOCK2, phospho-PI3Kp85α (Tyr 508), phospho-tyrosine (to detect activated PI3Kp101), p-ERK1/2, pan-ERK1/2, and GAPDH (Santa Cruz) were added to the membranes and incubated overnight at 4°C in 5% non-fat milk. Membranes were then washed and corresponding horseradish peroxidase (HRP) conjugated secondary antibodies (Santa Cruz) were added for 1 hour followed by additional washes. Immunoreactive proteins were visualized by a chemiluminescent detection reagent (Pierce) on autoradiographic films.

### Migration and invasion assay

Cell migration and invasion were assessed using BD Biocoat™ migration or Matrigel™ invasion chamber systems (BD Biosciences). Briefly, matrigel inserts were hydrated for 2 hours with 500 μl of DMEM at 37°C with 5% CO_2_. CXCL13 (100 ng/ml) was added to the bottom chamber containing serum-free RPMI medium. LNCaP and PC3 cells were pretreated with isotype control or anti-human CXCR5 antibody (1 μg/ml, R&D systems), G protein β and γ inhibitor (U-73122, 5 μM), wortmannin (1 μM), small molecule inhibitors of PI3Kp110α (PI-103, 3 μM), PI3Kp110β (TGX221, 1 μM), and PI3Kp110γ (AS605240, 3 μM), Src (SU6656, 5 μM), FAK (PF-573228, 5 μM), or DOCK2 siRNA prior to harvest, and added to the top chambers in serum-free RPMI medium at 10,000 cells per well. The cells were allowed to migrate or invade for 8 hours at 37°C with 5% CO_2_. Non-migrating cells on the upper surface of the membrane were removed with a cotton swab. The cells that migrated to the lower surface of the membrane were fixed with methanol at room temperature for 5 minutes, stained with crystal violet for 2 minutes, and washed with distilled water. The membranes were peeled and placed on glass slides. Cells were then counted by microscopy at 40× magnification and percent cell invasion was calculated as follows: percent invasion equals mean number of cells invading through Matrigel™ insert membrane divided by mean number of cells migrating through control insert membrane multiplied by 100. Experiments were performed in triplicate and repeated three times.

### Fast Activated Cell-Based ELISA (FACE) for ERK1/2

LNCaP and PC3 cell lines were cultured and seeded in 96-well plates at 5000 cells per well in complete RPMI supplemented with 10% FBS. Cells were serum-starved for 16 hours and pretreated with isotype control or anti-human CXCR5 antibody (1 μg/ml), pertussis toxin (100 ng/ml), G protein β/γ inhibitor (U-73122, 5 μM), wortmannin (1 μM), small molecule inhibitors of PI3Kp110α (PI-103, 3 μM), PI3Kp110β (TGX221, 1 μM), and PI3Kp110γ (AS605240, 3 μM), Src (SU6656, 5 μM), FAK (PF-573228, 5 μM), or DOCK2 siRNA. Following treatment with inhibitors or siRNA, cells were stimulated with (or without) CXCL13 (100 ng/ml) for 5 or 10 minutes. Next, FACE™ assays (Active Motif) were performed to measure modifications in the levels of p-ERK1/2 and total ERK1/2 expression by LNCaP and PC3 cell lines. Briefly, treated cells were fixed in 4% parafolmaldehyde in phosphate-buffered saline for 20 minutes. Antibody blocking buffer was added for 1 hour, followed by anti-p-ERK1/2- or total-ERK1/2 primary antibodies. Cells were then washed and HRP-conjugated secondary antibody was added for 1 hour. Chemiluminescence was read using a luminometer. After readings were recorded, cells were stained with crystal violet for 30 minutes and absorbance read on a spectrophotometer at 595 nm, which indicated the relative number of cells in each well. Experiments were performed in triplicate and results show the ratio of p-ERK1/2 to total ERK1/2 normalized to cell density per well.

### Statistical analysis

FACE™, migration, and invasion assays were performed in triplicate, and at least three separate studies with similar results were performed. Images from Western blotting or immunoprecipitation studies were visualized using VersaDoc Imaging System (Biorad) prior to densitometric quantitation. All data are presented as mean ± standard error. Statistical analysis was performed using paired or unpaired t tests as appropriate. A *p *value of < 0.05 was considered statistically significant.

## List of Abbreviations

ATCC: American Type Culture Collection; BCA: bicinchoninic acid; DOCK2: dedicator of cytokinesis 2; ERK1/2: extracellular signal-regulated kinase 1/2; FAK: focal adhesion kinase; FBS: fetal bovine serum; HRP: horseradish peroxidase; JNK-MAPK: Janus kinase - mitogen activated protein kinase; MMP: matrix metalloproteinase; PCa: prostate cancer; PI3K: phosphoinositide-3 kinase; PIP2: phosphoinositide 4,5-biphosphate; PIP3: phosphoinositide 3,4,5-triphosphate; PTEN: phosphatase and tensin homolog deleted on chromosome ten; RPMI: Roswell Park Memorial Institute; SDS-PAGE: sodium dodecyl sulfate-polyacrylamide gel electrophoresis; TTBS: Tris-Tween Buffered Saline.

## Competing interests

The authors declare that they have no competing interests.

## Authors' contributions

CH carried-out all experiments, quantified protein levels, and analyzed data with the assistance of PS, RS, PJ, JS, and SS. JL conceived the study, participated in its design with all authors, coordinated and helped to draft the manuscript with the assistance of all authors. All authors read and approved the final manuscript.
